# Unveiling the Biometric Potential of Finger-Based ECG Signals

**DOI:** 10.1155/2011/720971

**Published:** 2011-08-07

**Authors:** André Lourenço, Hugo Silva, Ana Fred

**Affiliations:** ^1^Instituto Superior de Engenharia de Lisboa, Scientific Area of Electronics, Telecommunications and Computers, 1959-007 Lisboa, Portugal; ^2^Instituto de Telecomunicações, Scientific Area of Networks and Multimedia, 1049-001 Lisboa, Portugal; ^3^Department of Electrical and Computer Engineering, Instituto Superior Técnico, 1049-001 Lisboa, Portugal

## Abstract

The ECG signal has been shown to contain relevant information for human identification. Even though results validate the potential of these signals, data acquisition methods and apparatus explored so far compromise user acceptability, requiring the acquisition of ECG at the chest. In this paper, we propose a finger-based ECG biometric system, that uses signals collected at the fingers, through a minimally intrusive 1-lead ECG setup recurring to Ag/AgCl electrodes without gel as interface with the skin. The collected signal is significantly more noisy than the ECG acquired at the chest, motivating the application of feature extraction and signal processing techniques to the problem. Time domain ECG signal processing is performed, which comprises the usual steps of filtering, peak detection, heartbeat waveform segmentation, and amplitude normalization, plus an additional step of time normalization. Through a simple minimum distance criterion between the test patterns and the enrollment database, results have revealed this to be a promising technique for biometric applications.

## 1. Introduction

As a biometric trait, electrocardiographic (ECG) signals have very appealing characteristics as they provide intrinsic liveliness detection and are strongly correlated to the subjects arousal level [1]. Therefore, the application of ECG for biometric purposes has been studied for long, both under controlled and unrestrained scenarios [2–5]. Recent work has shown the validity of the ECG signals for human identification [6–8].

While results enhance the potential of these signals, user acceptance may be limited by the data acquisition methods and apparatus. State-of-the-art research has revealed that, for biometric applications, a 1-lead setup suffices; nonetheless, a chest-mounted sensor apparatus with pregelled electrodes is typically used [9, 10]. Given this constraint, work in the field has begun to focus on ECG acquired at the finger tips.

In [11] a nonmedical data, acquisition setup is explored, which uses two electrodes connected at the subjects thumb tips; data acquisitions and performance evaluation were done for data collected within a group of 50 subjects. The authors process the collected signals for P-QRS-T segmentation and align the resulting waves to extract a mean wave. Classification results are obtained through the use of a distance metric based on wavelet coefficients, computed by doing a wavelet representation of the extracted mean waves.

We propose an ECG-based biometric system for human identification, that recurs to a minimally intrusive 1-lead setup for signal acquisition at the fingers. Our apparatus uses Ag/AgCl electrodes without gel as interface with the skin, further improving its usability. This work relies on time domain processing of the ECG signal. Due to the inherent heartbeat waveform variability, normalization must be performed in order to obtain amplitude and time invariant characteristics applicable for biometric purposes. The typical steps consist of filtering, peak detection, heartbeat waveform segmentation, and amplitude normalization; our approach further improves on prior work by adding an additional step of time normalization of the features.

The rest of the paper is organized as follows: Section 2 introduces an overview of the system and the proposed signal acquisition apparatus; Section 3 details the signal processing; Section 4 shows the experimental evaluation; finally, Section 5 outlines the main results and conclusions.

## 2. Data Acquisition

**Figure 1:**
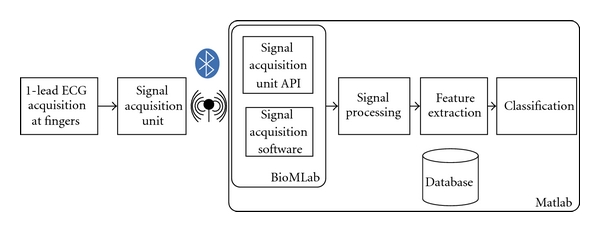
System architecture.

The system architecture is depicted in Figure 1. At the hardware level, we have the 1-lead ECG sensor setup connected to the signal acquisition unit, which transmits the data through a Bluetooth wireless connection to a base station (PC). At the base station, Matlab was used for data acquisition, processing, and storage. A specific API, BioMLab, was implemented to interface Matlab with the wireless acquisition unit, handling the low-level communication and signal acquisition tasks.

Signal processing and feature extraction blocks implement the signal analysis algorithms and feature extraction. Classification is performed using the features provided by the signal processing stage, and a database is used for data persistence. Also, a simple set of functions was implemented to handle the data storage and retrieval from the database. The database contains the set of features collected from each user during the enrollment.

### 2.1. Measurement Apparatus

Advances in biosignal sensors and data acquisition have led to wireless, wearable, and unobtrusive technologies for collecting ECG signals [12–14]. Still, current systems are mostly targeted at wellness and medical applications, requiring physical contact with the subjects body at the trunk and/or legs level. Furthermore, conductive paste or pregelled electrodes are generally required.

We propose a method and apparatus for ECG signal acquisition, through a single lead setup at the fingers, recurring to Ag/AgCl electrodes without gel. This setup intends to bring the usability and acceptability of ECG-based biometric systems to the level of other biometric traits, in terms of signal acquisition [15, 16].

**Figure 2:**
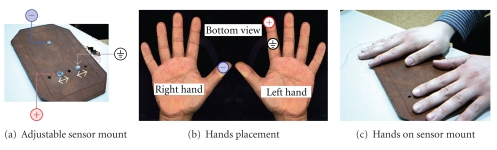
Signal acquisition setup.

Our adjustable sensor mount and measurement apparatus prototype is depicted in Figure 2. The rigid base, in Figure 2(a), integrates three leads which, due to the underlying sensor design, correspond to the ground, positive, and negative poles. The right hand thumb is used as negative electrode, and the left hand index finger simultaneously as the positive and ground electrodes, as illustrated in Figure 2(b).  Figure 2(c) illustrates the usage of the proposed setup.

The base sensor is an ecgPLUX [17] active ECG triode, and its specifications are listed in Table 1. The interface with the skin is done through dry Ag/AgCl electrodes without the application of any gel or conductive paste. For signal acquisition and transmission, we used a Bluetooth wireless bioPLUX [18] research biosignal acquisition unit.  Table 2 describes the main specifications of this system.

### 2.2. Heartbeat Waveform Segmentation

**Figure 3:**
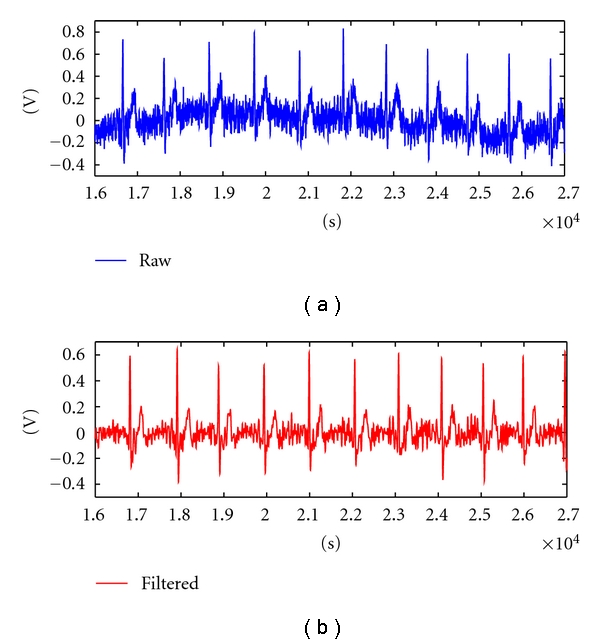
Sample of an ECG signal collected at the fingers (raw and filtered).

The first step consists of a band pass digital filtering of the signal, in the [0.5;30] Hz passing band using a FIR filter. These frequencies retain the necessary information for the proposed task while eliminating both the baseline wander and high-frequency noise.  Figure 3 shows an example of the signals acquired at the fingers using the proposed setup, where the existence of the different complexes can be easily observed.

The QRS detection is performed following an adaptation of the Englese and Zeelenberg algorithm [19], found to be one of the more robust for this purpose [20]. The filtered ECG signal is passed through a differentiator (1) and then by the sequence of filters ((2) and (3))

(1) $$\begin{array}{c} y_{0}\left[n\right]=x\left[n\right]-x\left[n-1\right], \end{array}$$

(2) $$\begin{array}{c} y_{1}\left[n\right]=y_{0}\left[n\right]-y_{0}\left[n-4\right], \end{array}$$

(3) $$\begin{array}{c} y_{2}\left[n\right]=\overset{4}{\underset{i=0}{\sum }}c_{i}\cdot y_{1}\left[n-i\right],\;\text{where}\;\;c_{i}=\left[1,4,6,4,1\right]. \end{array}$$

**Figure 4:**
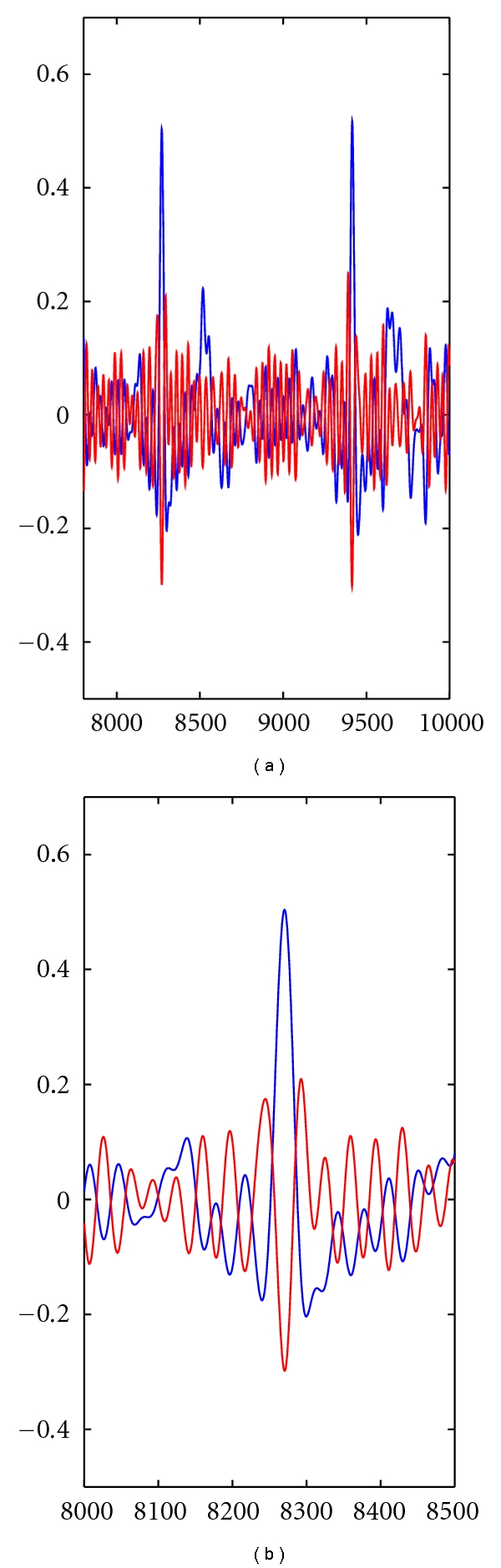
Peak detection using an adaptation of the Englese and Zeelenberg algorithm.

Figure 4 depicts the acquired signal, *x*[*n*] (in blue), and the filtered signal, *y*_2_[*n*] (in red). The presence of an *R* spike will induce a pronounced negative lobe and two positive lobes with lower amplitude in *y*_2_[*n*]. The detection algorithm is based on two thresholds masking the amplitude of these positive and negative lobes. Instead of using the ones proposed in [20], we calculated thresholds through experimental analysis of the data.

The detection of  “real” *R* spikes is concluded by computing the *RR* intervals based on neighbor *R* spike and using an additional verification based on reference physiological limits of these intervals [21]. We consider as valid *R* peaks, the ones whose neighbor *R* peaks rhythm is within the interval [*minLatency*, *maxLatency*], where *minLatency* corresponds to 150 BPM and *maxLatency* to 30 BPM.

After computing the *R* peaks, we continue with the segmentation of the ECG signal, identifying the *Q* and *S* complexes. For the identification of these complexes we continue to use *y*_2_[*n*].

Taking as reference the identified *R* peak, we analyze the *y*_2_[*n*] signal within its neighborhood, determining the time instants were it starts to be positive and comes down to negative again, determining the intervals [*iStartQ*, *iEndQ*] and [*iStartS*, *iEndS*]. Within these intervals, we take the minimum value of *x*[*n*] as the *Q* and *S* complexes. The final step for determining the heartbeat waveform is finding the *P* and *T* complexes.

For the *P* complex, we look for the maximum value of *x*[*n*] in the interval [*leftMostIndex, iStartQ*], where the *leftMostIndex* was determined as the *R* peak time, subtracted by the typical PQR latency interval upper bound. For the determination of the *T* complex, we follow a similar process, finding the maximum value of *x*[*n*] in the interval [*iEndS, rightMostIndex*], where *rightMostIndex* was determined as the *R* spike time plus the typical RST latency upper bound.

We consider as valid P-QRS-T complexes, sequences of signals, where (a) *P* and *T* peak values are higher than zero amplitude; (b) the *P* complex starts at least within 30 ms before the *Q* complex.

## 3. Signal Processing and Feature Extraction

**Figure 5:**
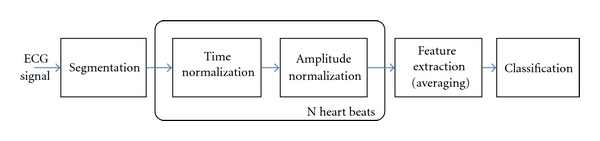
Signal processing and feature extraction block diagram.

After heartbeat segmentation, we obtained a sequence of the P-QRS-T complexes.  Figure 5 illustrates the remaining signal processing and feature extraction steps. The rational behind ECG biometrics is that the heartbeat wave form is different from subject to subject; nonetheless, heartbeat cycles vary in length and amplitude. This may occur not only between subjects but also for the same subject in different moments of time, a reason for which our approach seeks to obtain a latency and amplitude invariant set of features. We proceed with a time and amplitude normalization, rescaling each segment to the same number of points and amplitude. Finally, we extract features from the normalized signals.

### 3.1. Time Normalization

**Figure 6:**
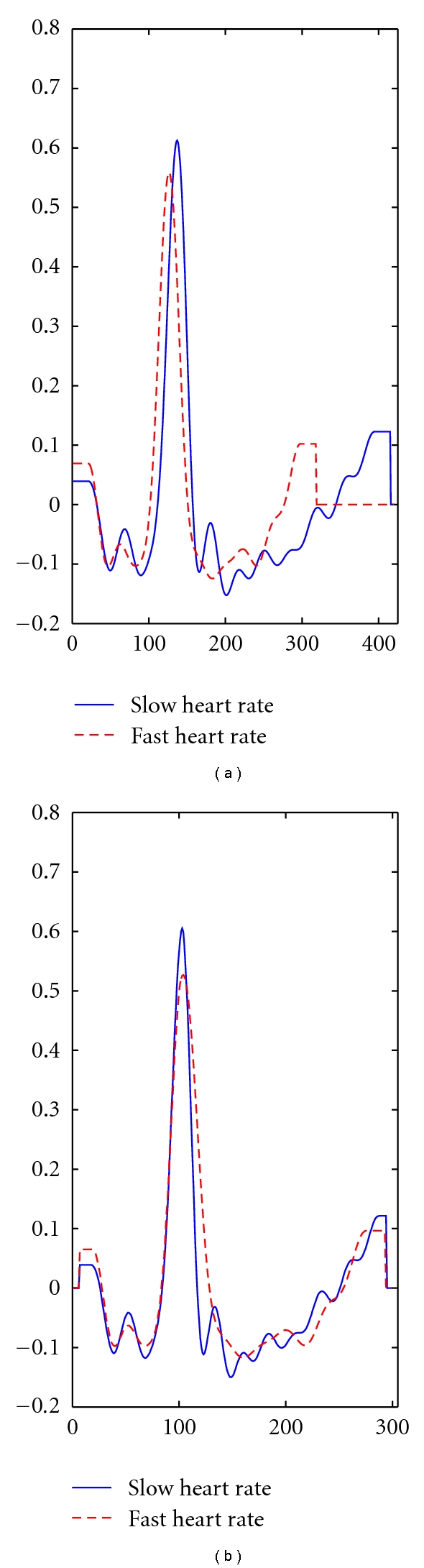
Comparison of heartbeat waveforms at heart rates of 70 (low) and 133 (high) BPM. On the left we depict the raw signals, where, a wave compression can be noticed for high heart rate. On the right, the time-normalized signals are presented.

Changes in the heart rate typically result in the time compression/expansion of the heartbeat waveform. The normalization of the segmented heartbeat signal will ensure that the variability of the latencies of each complex is reduced.  Figure 6 illustrates one example of an acquisition where the subject presented a computed heart rate varying from 133 to 70 beats per minute (BPM), from the beginning to the end of the acquisition, showing the expansion/compression effect on the waveform caused by different heart rate values.

Usually, the normalization of the segmented signals is performed decimating the signal in between a fixed window centered around the *R* peaks. In this work, we followed a nonuniform decimation procedure which does not use fixed time windows, but the ECG signal fiducial points themselves. This procedure is divided in two parts: decimation of the interval between the beginning of the *P* complex until the *R* peak; decimation of the interval between the *R* peak and the end of the *T* complex.

The devised algorithm samples these intervals so that each pattern has the same number of samples regardless of the expansion/compression of the heartbeat waveforms. The resulting normalized signals will all have the same number of samples and the *R* peak at the same time instant. In this study, we use 300 samples for each single heartbeat.

### 3.2. Amplitude Normalization

The ECG signal processing is only concluded with the amplitude normalization step. We take the segmented time-normalized signals and normalize them using as normalization factor the average of the amplitude of the obtained *R* peaks. This value normalizes the intrasubject amplitude difference, reducing differences in amplitude that can happen during one acquisition.

**Figure 7:**
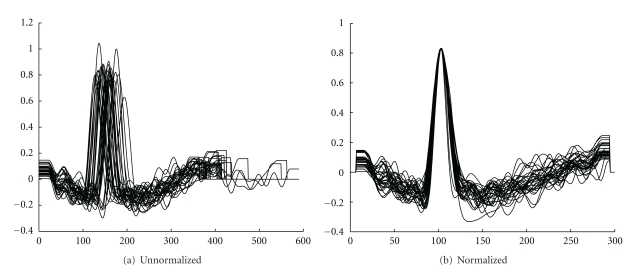
Example of unnormalized and normalized segmented signals.

Figure 7 illustrates an example before and after time and amplitude normalization, for signals obtained during one acquisition.

### 3.3. Feature Extraction

In the literature, there are several approaches for ECG feature extraction: fiducial [3–5, 8] and nonfiducial [6, 11]. Fiducial methods use points of interest within a single heartbeat waveform, such as local maxima or minima; these points are used as reference to allow the definition of latency times. Several methods exist that extract different time and amplitude features, using these reference points. Nonfiducial techniques aim at extracting discriminative information from the ECG waveform without having to localize fiducial points.

In this work, we compute a single mean heartbeat, averaging all the normalized signals. The features are directly the amplitudes of this waveform. This approach contrasts with previous works [6, 8], where the mean wave was computed for every 10 consecutive segmented heartbeat waveforms, and were fiducial points where extracted. The templates were composed by the concatenation of the features extracted from each mean wave, therefore increasing the spacial complexity.

Nevertheless, our approach can be considered fiducial, since the normalized signals are obtained based on a segmentation that depends on the location of the P-QRS-T complexes.

## 4. Experimental Results

For the evaluation of the system, we populated a database with acquisitions from 16 subjects. For each user, we collected 2 minutes of ECG signals at the fingers using the proposed apparatus. Classification was performed using a minimum Euclidean distance criterion between the test templates and the enrollment templates (1-NN classifier).

The systematic evaluation of the system is based on cross validation, using 30 runs of enrollment/test sequence for each user. For the enrolment, we randomly select 30 single heartbeats, averaging them to form the enrolment template; for the test, we also select 30 single heartbeats (different from the previous), averaging them to construct test templates. Results are computed from the average of the 30 runs.

### 4.1. Identification

**Figure 8:**
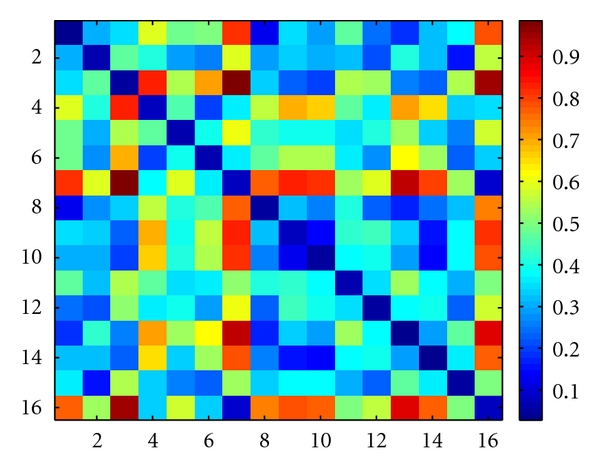
Distance matrix between enrolment and test templates.

Figure 8, presents the distance matrices obtained with the proposed methodology between enrolment and test templates. The element *i*, *j* of the matrix represents the distance from the subject *i* to the subject *j*, according to the selected set of features. In the presented color scheme, blue is attributed to values close to zero, representing subjects with very similar features, and red is attributed to values close to one, representing subjects very dissimilar.

**Figure 9:**
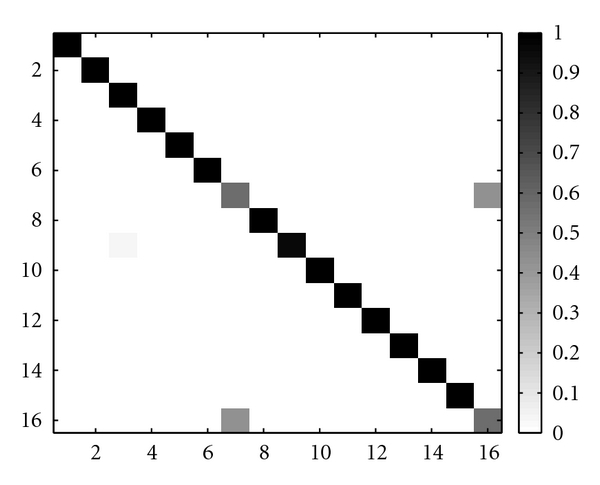
Confusion matrix.

In the matrix of Figure 8 we see that there are very few entries with blue color, except in the diagonal, which represents the distance from the subject to himself. This characteristic is important in order to have a high true positive rate (TPR). Following a minimum distance criterion between the test patterns and the enrollment templates, we obtain as decision the matrix found in Figure 9, corresponding to an identification accuracy of 94,3%.

### 4.2. Authentication

**Figure 10:**
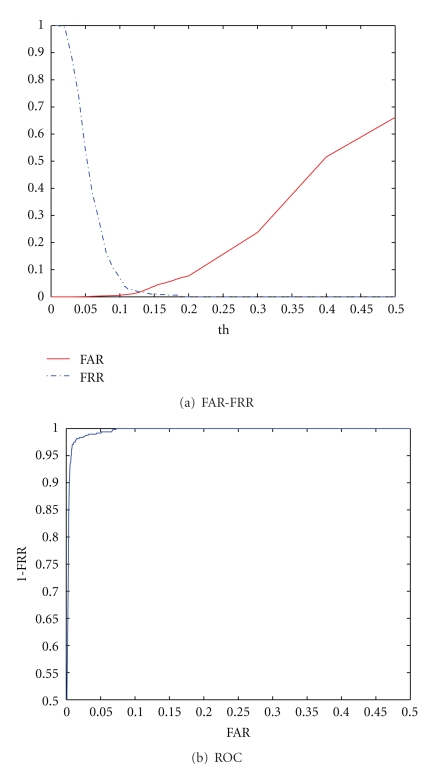
FAR-FRR and ROC curves.

In an authentication scenario, an individual is accepted if the euclidean distance between the enrolment template and the test template is inferior to a given threshold (*th*).  Figure 10 summarizes the performance of the proposed system in an authentication scenario, showing the False Acceptance Rate (FAR) versus False Rejection Rate (FRR) and the ROC curves. The obtained equal error rate (EER) is 13,0%.

To obtain further improvements on the authentication performance, we evaluated a user-tuned threshold selection method. Using this approach, in each test run, the FAR and FRR are computed per subject, and, from these, the individual EER and optimal decision threshold are determined. Over all runs, the average EER over all users was improved to 10,1% using this approach.

### 4.3. Discussion

The obtained results outperform state-of-the-art results on identifications based on ECG acquired on fingers. In [11], a classification accuracy of 89% over a population of 50 subjects during three data-recording sessions on different days is reported. One of the limitations of our study is the database size, composed by 16 subjects, and also by the fact that only one data-recording session was performed. The testing variability was accomplished through the use of cross-validation, with random sampling of enrollment and test heartbeats on each run.

Compared with state-of-the-art results on ECG signals acquired on chest (lead V2), using the same type of measurement apparatus, 1-lead pregelled electrodes, our results are slightly worst, as reported classification results reach 100% accuracy on identification. Such a difference could be related to the quality of the acquired signal, as the signal acquired from the fingers has significantly lower signal-to-noise ratio. This can be caused by factors as the lower conductance on the electrode-skin interface, higher sensitivity to external electromagnetic interference, and different signal processing methodologies.

Future work will be focused on improving the signal-to-noise ratio of our signals. Our next steps will target on improving directly the acquisition apparatus, through the development of a dedicated sensor with custom specifications. Moreover additional research will be performed regarding signal processing of the acquired signal, seeking further improvements in terms of noise and potential outliers removal from the segmented heartbeats.

## 5. Conclusions

This paper describes a methodology and apparatus for human biometric identification and authentication based on 1-lead ECG signals collected at the fingers. Our goal was to provide the building blocks for a nonintrusive ECG-based biometric system.

We have devised a measurement apparatus that only requires slight contact with the subject hands without the need of pregelled electrodes or conductive paste, providing a signal acquisition setup similar to the ones already used by other, largely accepted, biometric traits.

Experimental evaluation has been performed on a group of 16 subjects, from which the signals were collected at the fingers, and promising results were revealed. The proposed approach allowed us to obtain a 94,3% recognition rate in subject identification and a 13,0% EER in subject authentication. By applying a user-tuned threshold selection method, authentication results were further improved to a 10,1% EER.

Future work will focus on extending the subject base and experimenting alternative feature analysis and classification methods, targeting a continuous real-time biometric system.

## Figures and Tables

**Table 1:** ecgPLUX sensor specifications.

Gain	1000
Filtering	0.05–30 Hz
CMRR	110 dB
Input impedance	>1 MOhm

**Table 2:** bioPLUX research specifications.

Connectivity	Bluetooth class II
Sampling rate	1000 Hz
Channels	8 An. + 1 Dig.
Size	84 × 53 × 18 mm
Weight	86 g
